# Astragaloside IV Inhibits Adipose Lipolysis and Reduces Hepatic Glucose Production *via* Akt Dependent PDE3B Expression in HFD-Fed Mice

**DOI:** 10.3389/fphys.2018.00015

**Published:** 2018-01-23

**Authors:** Qun Du, Shuihong Zhang, Aiyun Li, Imran S. Mohammad, Baolin Liu, Yanwu Li

**Affiliations:** ^1^Pi-Wei Institute, Guangzhou University of Chinese Medicine, Guangzhou, China; ^2^Jiangsu Key Laboratory of TCM Evaluation and Translational Research, Department of Pharmacology of Chinese Materia Medica, China Pharmaceutical University, Nanjing, China; ^3^Experiment Center for Science and Technology, Shanghai University of Traditional Chinese Medicine, Shanghai, China; ^4^Department of Pharmaceutics, China Pharmaceutical University, Nanjing, China

**Keywords:** astragaloside IV, Akt, PDE3B, lipolysis, gluconeogenesis

## Abstract

**Objective:** This study aims to investigate the effect of astragaloside IV on adipose lipolysis and hepatic gluconeogenesis.

**Methods:** High-fat diet (HFD) feeding induced adipose dysfunction with enhanced endogenous glucose production in mice. The effects of Astragaloside IV on lipolysis and hepatic glucose production were investigated.

**Results:** HFD feeding induced cAMP accumulation through reducing PDE3B expression and activity in adipose tissue. As a result, HFD feeding increased adipose lipolysis in mice. Astragaloside IV enhanced Akt phosphorylation and promoted Akt binding to PDE3B to preserve PDE3B content, resultantly reducing adipose cAMP accumulation. Knockdown of Akt1/2 diminished the effect of astragaloside IV on PDE3B induction, indicative of the role of Akt in astragaloside IV action. As a result from blocking of cAMP/PKA signaling, astragaloside IV suppressed hormone-sensitive lipase (HSL) activation and inhibited inflammation-associated adipose lipolysis. Moreover, astragaloside IV reduced ectopic fat deposition in the liver and inhibited FoxO1 activation *via* regulation of Akt, resultantly restraining excess hepatic glucose production.

**Conclusion:** We showed that preserving PDE3B content by Akt is a key regulation to prevent lipolysis. Astragaloside IV inhibited lipolysis by reducing cAMP accumulation *via* regulation of Akt/PDE3B, contributing to limiting hepatic lipid deposition and restraining excessive hepatic glucose production.

## Introduction

In obesity, the adipose tissue frequently represents inflammation, endoplasmic reticulum (ER) stress and dysregulation of adipokine expression, which contribute to diabetes and insulin resistance through various mechanisms (Yuan et al., 2001; Greenberg and Obin, 2006; Wang et al., 2016). Adipose tissue serves as a site for fat storage, where fatty acids stored as triacylglycerols in adipocytes, constitute the primary energy reserves. Meanwhile, the stored triacylglycerols could be hydrolyzed by lipases, such as adipose triglyceride lipase (ATGL) and HSL, leading to the release of glycerol and free fatty acids (FFAs). The increased free FFAs flux from adipose tissue to non-adipose tissue promotes ectopic fat deposits and increases lipid accumulation in liver and skeletal muscle, leading to insulin resistance (Perry et al., 2015; Ritter et al., 2015; Wang et al., 2016). Dysregulation of adipose lipolysis increases the release of FFAs into the circulation, and consistent with this, elevated levels of circulating FFAs are often observed in individuals subjected to insulin resistance and diabetes (Coppack et al., 1992).

It was noted that, the several hormones and effectors can induce lipolysis in adipose tissue by the activation of cAMP-dependent protein kinase A (PKA). As a second messenger for cellular responses, cAMP is formatted by adenylate cyclase and constantly degraded by phosphodiesterase (PDEs). In response to cAMP accumulation, PKA activation initiates lipolysis cascade through activation of HSL and perilipin, which break triglycerides (TGs), resulting in the release of glycerol and FFAs (Belfrage et al., 1980; Strålfors and Belfrage, 1983). As cAMP signaling is located at the upstream of lipolysis cascades, the regulation of cAMP is a key determinant in the control of the downstream lipolysis process. PDEs are the enzymes that degrade cAMP or cGMP by breaking the phosphodiester bond and PDE3B is proposed to be the predominant isoform of PDEs in adipose tissue, capable of cAMP degradation (Nilsson et al., 2006; Degerman et al., 2011). Insulin inhibits lipolysis and regulation of PDE3B is a key step in its action (Kitamura et al., 1999; Frühbeck et al., 2014). In contrast, proinflammatory cytokine TNF-α increases cAMP accumulation by suppression of PDE3B, and thus induces lipolysis (Rahn Landström et al., 2000; Zhang et al., 2002), indicative of the involvement of inflammation in lipolysis.

Astragaloside IV is a saponin and main active component in the medicinal plant *Astragalus membranaceus*, which widely used in traditional Chinese medicine for the treatment of metabolic disorders. Astragaloside IV exerts anti-diabetic effects (Lv et al., 2010), improves metabolic parameters in fructose-fed mice and ameliorates adipose dysfunction (Jiang et al., 2008; Zhang et al., 2011), well-demonstrating its action in the improvement of metabolism. It is generally accepted that insulin inhibits lipolysis through PI3K/Akt signaling (Kitamura et al., 1999). However, this knowledge has been challenged by recent published studies, which showed that Akt is dispensable for insulin to suppress lipolysis (Frühbeck et al., 2014; Koren et al., 2015). Similarly, Akt is also proposed to be dispensable for insulin to suppress hepatic gluconeogenesis (Lu et al., 2012; Titchenell et al., 2015). These findings raise question whether pharmacological activation of Akt could inhibit adipose lipolysis and hepatic glucose production is independent on insulin. Astragaloside IV protects cardiac function through regulation of PI3K/Akt and Akt/GSK-3β signaling, indicative of its positive effect on Akt activation (He et al., 2012; Jia et al., 2014). Therefore, it is tempting to know if astragaloside IV could inhibit adipose lipolysis *via* regulation of Akt. To address this issue, we investigated the effect of astragaloside IV on lipolysis in the adipose tissue of high-fat diet (HFD)-fed mice and found that astragaloside IV could inhibit lipolysis by reducing cAMP accumulation *via* regulation of Akt/PDE3B, contributing to limiting hepatic lipid deposition and restraining excessive hepatic glucose production. This finding provides novel mechanistic insights regarding the protective effects of astragaloside IV on metabolic homeostasis and contributes to the design of new strategies for the management of metabolic diseases.

## Materials and methods

### Materials

Astragaloside IV (purity ≥ 98%) was obtained from Shanghai Forever Biotech Co., Ltd. (Shanghai, China). Astragaloside IV was dissolved in DMSO to prepare 10 mM stock solution and then was diluted at 1,000-fold in culture medium to generate a working concentration at 10 μM. 0.1% DMSO as a solvent control was run concurrently with the experiments. Palmitate (PA, Sinopharm, Shanghai, China) was dissolved in ethanol to prepare 200 mM stock solution and then further diluted with medium containing 10% FFA-free BSA at the ratio of 1:19 to obtain a concentration of 10 mM before use. Metformin was obtained from Sino-American Shanghai Squibb Pharma (Shanghai, China). Akt inhibitor triciribine, MK2206 and AZD5363 was from Apex Bio (Houston, USA). Isoproterenol was from Shanghai Harvest Pharmaceutical Co., Ltd. (Shanghai, China). TNF-α was from R&D Systems.

### Animals

Male ICR mice (6–8 weeks of age) were purchased from the Laboratory Animal Center of Nanjing Qinglongshan and were acclimatized in facility with a constant temperature (22 ± 1°C) and a 12-h light-dark cycle with free access to water and food. All experiments were approved by Animal Ethics Committee of China Pharmaceutical University.

Mice were fed with normal chow diet or HFD containing 10% lard, 10% yolk, 1% cholesterol, 0.2% cholate, and 78.8% standard diet simultaneously with oral administration of astragaloside IV (50, 100 mg/kg) or metformin (200 mg/kg) daily for 2 weeks. While, control mice were received the vehicle only. Mice body weight and food intake were measured and recorded daily. Two weeks later, after 12 h fasting, blood samples were collected from orbital sinus to examine total cholesterol (TC), TG, glucose, FFAs, and glycerol concentrations in the serum using commercial kits. Meanwhile, the epididymis adipose tissue was rapidly isolated and stored at −80°C for further assay.

### Cell culture

3T3-L1 cells (a cell line of preadipocytes, Cell Bank of Chinese Academy of Sciences, Shanghai, China) were grown in Dulbecco's Minimum Essential Medium (DMEM, Gibco, USA) containing 10% FBS, 100 μg/mL of streptomycin and 100 U/mL of penicillin. At 80–90% of confluence, the culture medium were replaced with fresh DMEM supplemented with 10% FBS, isobutylmethylxanthine (0.5 M), dexamethasone (1 μM), and insulin (10 μg/mL) for next 48 h. Then, change the medium with DMEM containing 10% FBS and insulin (10 μg/mL) for another 8–10 days for differentiation.

### Measurement of glycerol and FFAs release

The normal mice or the HFD-fed mice were sacrificed by cervical dislocation. The epidydimal adipose tissue was isolated and chopped into small pieces immediately. After incubation in DMEM for 24 h, FFAs and glycerol concentrations in the medium were determined by commercial kits (Jiancheng Bioengineering Institute, Nanjing, China). The contents of FFAs and glycerol in the serum were also determined with the commercial kits (Jiancheng Bioengineering Institute, Nanjing, China) according to the manufacturers' instructions. For isoproterenol or TNF-α stimulation, the epididymis adipose tissue of normal mice was stimulated with isoproterenol (1 μM) or TNF-α (20 ng/mL) for 2 or 16 h, respectively, in the presence of indicated agents.

### Determination of cAMP, AMP, and cytokines in the adipose tissue

Adipose tissue were rinsed and homogenized in cold lysis buffer to prepare 10% adipose homogenate. After centrifuged at 12,000 g for 15 min at 4°C, the supernatant was collected to measure the concentration of cAMP (USCN, Wuhan, China) and AMP (Chengbin Biotech, Shanghai, China) using the corresponding commercial assay kits, while TNF-α and IL-6 contents were measured using ELISA Kits (Cusabio Biotech, Wuhan, China), respectively. For isoproterenol treatment, the isolated adipose tissue of normal mice was incubated with isoproterenol (1 μM) for 2 h in the presence of indicated agents.

### Measurement of PDE activity in adipose tissue

Epididymis adipose tissue was isolated from HFD-fed mice and homogenized in double-distilled water. The supernatant was desalted by gel filtration and then measured using a PDE activity assay kit (Colorimetric) (ab139460; Abcam, Cambridge, MA).

### Determination of hepatic triglyceride and acetyl CoA

The liver was isolated from HFD-fed mice and homogenized in ice-cold RIPA lysis buffer. The homogenates were then centrifuged and the supernatants was collected for the detection of TG and acetyl CoA concentrations using commercial kits (Shuojia, Shanghai, China) according to the manufacturer's instructions.

### Glucose and pyruvate tolerance tests

For pyruvate or glucagon tolerance test, fasted HFD-fed mice were treated with oral administration of astragaloside IV (50, 100 mg/kg) or metformin (200 mg/kg). Then after 2 h, mice were intraperitoneally injected with pyruvate (2 g/kg) for pyruvate tolerance test, or orally administrated with glucose (2.0 g/kg) for glucose tolerance test. Blood samples were collected from the orbital sinus at regular intervals for the assay of glucose using a commercial Kit. Blood glucose area under curve (AUC) was calculated as the follows: 0.5 × [Bg0 + Bg0.5]/2 + 0.5 × [Bg0.5 + Bg1.0]/2 + [Bg1 + Bg2]/2 (Bg0, Bg0.5, Bg1.0, and Bg2.0 referred to the blood glucose content at 0, 0.5, 1.0, and 2.0 h).

### Hepatic glucose production

The fasted mice (over 18 h) were sacrificed by cervical dislocation and liver was perfused in situ with Hank's Balanced Salt Solution, followed by digestion with collagenase IV. The collected hepatocytes were resuspended and cultured for further treatment in DMEM supplemented with 10% FBS and incubated at 37°C in an atmosphere of 5% CO_2_. For glucose production, hepatocytes were pretreated with indicated treatment and cultured in glucose-free media supplemented with 10 mM pyruvate for 6 h. Then the Glucose contents in the medium were assayed.

### Small interfering RNA transfection

3T3-L1 cells were cultured with small interfering RNA (siRNA) duplexes specific Akt (Santa Cruz Biotechnology, CA, USA) or a noncoding siRNA using transfection reagents for 7 h. After the transfection, the culture medium was replaced with DMEM containing 10% FBS for 48 h for experiments.

### Immunoprecipitation

For immunoprecipitation, differentiated 3T3-L1 cells were pre-treated with astragaloside IV (10 μM) for 0.5 h and then incubated with 100 μM of PA for 4 h. Then, adipocytes were washed and lysed on ice for 15 min. Next, the lysate was centrifuged at 12,000 g for 20 min and the cleared fractions were collected. Cleared fractions were incubated with anti-PDE3B antibody overnight at 4°C, following by incubation with protein A+G agarose beads (Beyotime Institute of Biotechnology, Shanghai, China) for 2 h. After washed five times with the lysis buffer, immunoprecipitates were boiled in 1% SDS loading buffer and used to be immunoblotted. For immunoprecipitation of the liver of HFD mice, the protein was collected and immunoprecipitated by using anti-FoxO1 antibody (Cell Signaling, Danvers, MA).

### Western blot analysis

Total proteins were extracted from the adipose tissue, liver or adipocytes using cold RIPA buffer (Beyotime Institute of Biotechnology, Nanjing, China) with 1% phenylmethanesulfonyl fluoride. Protein concentration was determined using a Pierce™ BCA Protein Assay Kit (Beyotime Institute of Biotechnology, Nanjing, China). To analysis the expression of protein, aliquots containing 60 μg of protein were electrophoresed by SDS-PAGE, then transferred to a polyvinylidene fluoride membrane. After blocking with TBST supplemented with 5% nonfat milk powder at room temperature for 2 h, membranes were incubated with primary antibodies, including PDE3B (Bioworld Technology, St. Paul, MN, USA), p-PKA substrate (Cell Signaling Technology, Beverly, MA, USA), p-HSL (S565–S660) (Cell Signaling Technology), HSL (Cell Signaling Technology), p-JNK(Bioworld Technology), JNK (Bioworld Techology), p-Akt (S473) (Cell Signaling Technology), Akt (Cell Signaling Technology), PAS (Cell Signaling Technology), p-PDH (E1-alpha) (phospho S293) (Abcam), PDH (Abcam), PC [EPR7365] (Abcam), and GAPDH (Bioworld Techology) for overnight at 4°C. Then, the membranes were washed for three times and incubated with the secondary antibody at room temperature for 2 h. The immunoblots were visualized by ECL Western Blot Detection System and quantized by Image-ProPlus 6.0 software. The relative intensities of each band were calculated by the mean intensity of four independent experiments.

### Quantitative real-time PCR

Total RNA was isolated from the liver or adipose of HFD-fed mice. RNA obtained was reversely transcribed into cDNA by using the HieffTM First Strand cDNA Synthesis Super Mix for RT-qPCR+gDNA wiper system (Yeasen). The relative gene expression was relatively quantified by HieffTM qPCR SYBR Green Master Mix (No Rox Plus) kit (Yeasen) with CFX96TM realtime system (BIO-RAD, USA). Primer sequences were shown below: PEPCK, F: GTGCTGGAGTGGATGTTCGG, R: CTGGCTGATTCTCTGTTTCAGG; G6PaseF: ACTGTGGGCATCAATCTCCTC, R: CGGGACAGACAGACGTTCAGC; PDE3B, F: AAAGCGCAGCCGGTTACTAT, R: CACCACTGCTTCAAGTCCCAG; β-actin, F: GGGAAATCGTGCGTGAC, R: AGGCTGGAAAAGAGCCT. The results of mRNA level were calculated with the 2^−ΔΔCt^ method.

### Immunofluorescence

After treatment, differentiated 3T3-L1 cells were fixed with 4% paraformaldehyde for 20 min, and permeabilized with 0.2% Triton X-100 for 10 min at room temperature. After blocking with 3% BSA, specimens were then labeled with specific primary antibodies (anti-PDE3B, anti-p-Akt) overnight at 4°C, followed by incubation with fluorescent secondary antibodies for 1 h at 37°C. After washing, specimens were examined with a confocal scanning microscope (Zeiss LSM 700).

### Statistical analysis

All data were expressed as mean ± SD. Results were analyzed using Student's *t*-test and one-way analysis of variance (ANOVA) followed by Student-Newman-Keuls multiple comparison test. Values of *p* < 0.05 were considered statistically significant.

## Results

### Astragaloside IV prevented lipolysis in adipose tissue of HFD-fed mice

**Figure 1:**
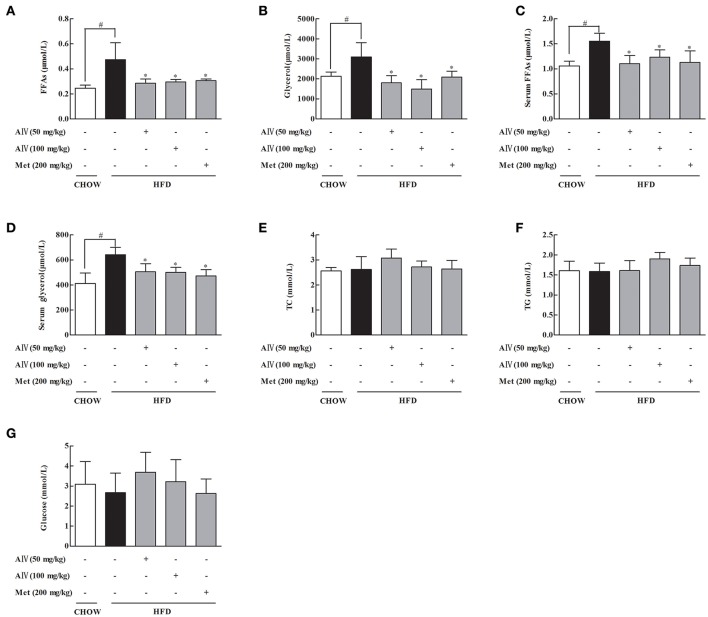
Astragaloside IV prevented lipolysis in adipose tissue of HFD-fed mice. Mice were fed with high fat diet (HFD) for 2 weeks and simultaneously administrated with astragaloside IV (AIV, 50 and 100 mg/kg) and metformin (Met, 200 mg/kg) by gavage. **(A,B)**, Free fatty acids (FFAs) and glycerol contents in the adipose tissues; **(C,D)**, Free fatty acids (FFAs) and glycerol contents in the blood. **(E–G)**, Total cholesterol (TC), triglyceride (TG), and glucose in the blood. Data were expressed as the mean ± SD (*n* = 6). ^*^p < 0.05 vs. model; ^#^*p* < 0.05 vs. control.

To investigate the effects of astragaloside IV on adipose dysfunction, we first observed the regulation of lipolysis in the freshly isolated epididymal adipose tissue from HFD-fed mice, and found that oral administration of astragaloside IV at concentrations of 50 and 100 mg/kg significantly reduced FFAs and glycerol release (Figures 1A,B). Similar to astragaloside IV, anti-diabetic agent metformin also reduced FFAs, and glycerol release from adipose tissue (Figures 1A,B). Consistent with what observed in adipose tissue, astragaloside IV, and metformin effectively reduced the elevated levels of blood FFAs and glycerol in HFD-fed mice (Figures 1C,D), while the contents of TC, triacylglycerol and glucose in the blood were not affected (Figures 1E–G). These results showed that astragaloside IV inhibited lipolysis in HFD-fed mice.

### Astragaloside IV reduced cAMP accumulation in adipose tissue

**Figure 2:**
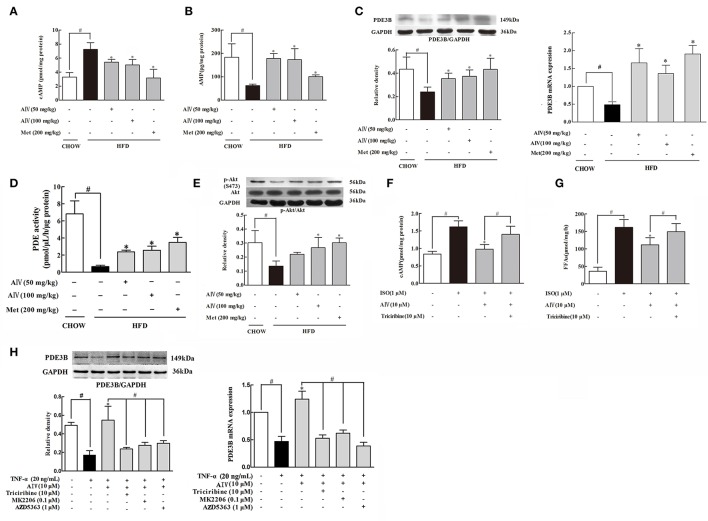
Astragaloside IV reduced cAMP accumulation in adipose tissue. **(A,B)**, The contents of cAMP and AMP in the adipose tissues of HFD-fed mice (*n* = 6); **(C)**, PDE3B gene and protein expression in the adipose tissue of HFD-fed mice was examined by Western blot (*n* = 4); **(D)**, PDE activity in the adipose tissue of HFD-fed mice was measured using a PDE activity assay kit (*n* = 6); **(E)**, The phosphorylation of Akt in the adipose tissue of HFD-fed mice was examined by Western blot. The epididymal adipose tissue of normal mice was incubated with isoprenaline (ISO) in the presence of astragaloside IV (AIV) or Akt inhibitor triciribine for 2 h. The contents of cAMP **(F)** and FFAs **(G)** were assayed by commercial kits (*n* = 6); **(H)**, Isolated epididymal adipose tissue was pretreated with astragaloside IV (AIV) or three Akt inhibitors triciribine, MK2206 and AZD5363, and then stimulated with TNF-α for 16 h. PDE3B gene and protein expression was detected (*n* = 4), (Astragaloside IV, AIV; Metformin, Met). The results were expressed as the mean ± SD. ^*^p < 0.05 vs. model; ^#^*p* < 0.05 vs. control.

As a second messenger, cAMP is a key regulator responsible for the initiation of lipolysis in adipose tissue. HFD feeding induced cAMP accumulation in adipose tissue along with the reduction of AMP levels, whereas astragaloside IV as well as metformin reversed the alterations in adipose tissue (Figures 2A,B). PDE3B, a member of PDEs, predominantly expresses in the adipose tissue, exerting the ability to degrade cAMP. We observed that HFD feeding significantly attenuated PDE3B gene and protein expression and meanwhile inhibited PDE enzymatic activity (Figures 2C,D) in adipose tissue. Astragaloside IV as well as metformin normalized PDE3B gene and protein expression and preserved PDE enzymatic activity in the adipose tissue of HFD-fed mice, suggesting the possibility to prevent cAMP accumulation by preserving PDE3B content by promoting its gene expression, at least in part.

Akt activation is documented to regulate PDE3B activity (Kitamura et al., 1999). In the adipose tissue, astragaloside IV prevented HFD-induced Akt impairment by preserving phosphorylation (Figure 2E). We isolated epididymal adipose tissue from normal mice and treat with isoproterenol to measure cAMP contents and FFAs release. We noted that astragaloside IV significantly decreased isoproterenol-induced cAMP and reduced FFAs release in isolated adipose tissue, but this action was blocked by co-treatment with Akt inhibitor triciribine (Figures 2F,G). Moreover, pro-inflammatory cytokine TNF-α impaired PDE3B both gene and protein expression in adipose tissues (Figure 2H), indicative of the involvement of inflammation in lipolysis. Similarly, the protective effect of astragaloside IV on PDE3B gene and protein expression was also abolished by Akt inhibitor triciribine (Figure 2H). To avoid unexpected off-target effects, we chose another two specific Akt inhibitors: MK2206 and AZD5363 and observed that both of this two inhibitors could abolished the protective effect of astragaloside IV on PDE3B gene and protein expression (Figure 2H), further confirming the involvement of Akt on PDE3B regulation. These results suggested that Akt was involved in Astragaloside IV action to prevent cAMP accumulation in adipose tissue.

### Astragaloside IV preserved PDE3B content *via* regulation of akt

**Figure 3:**
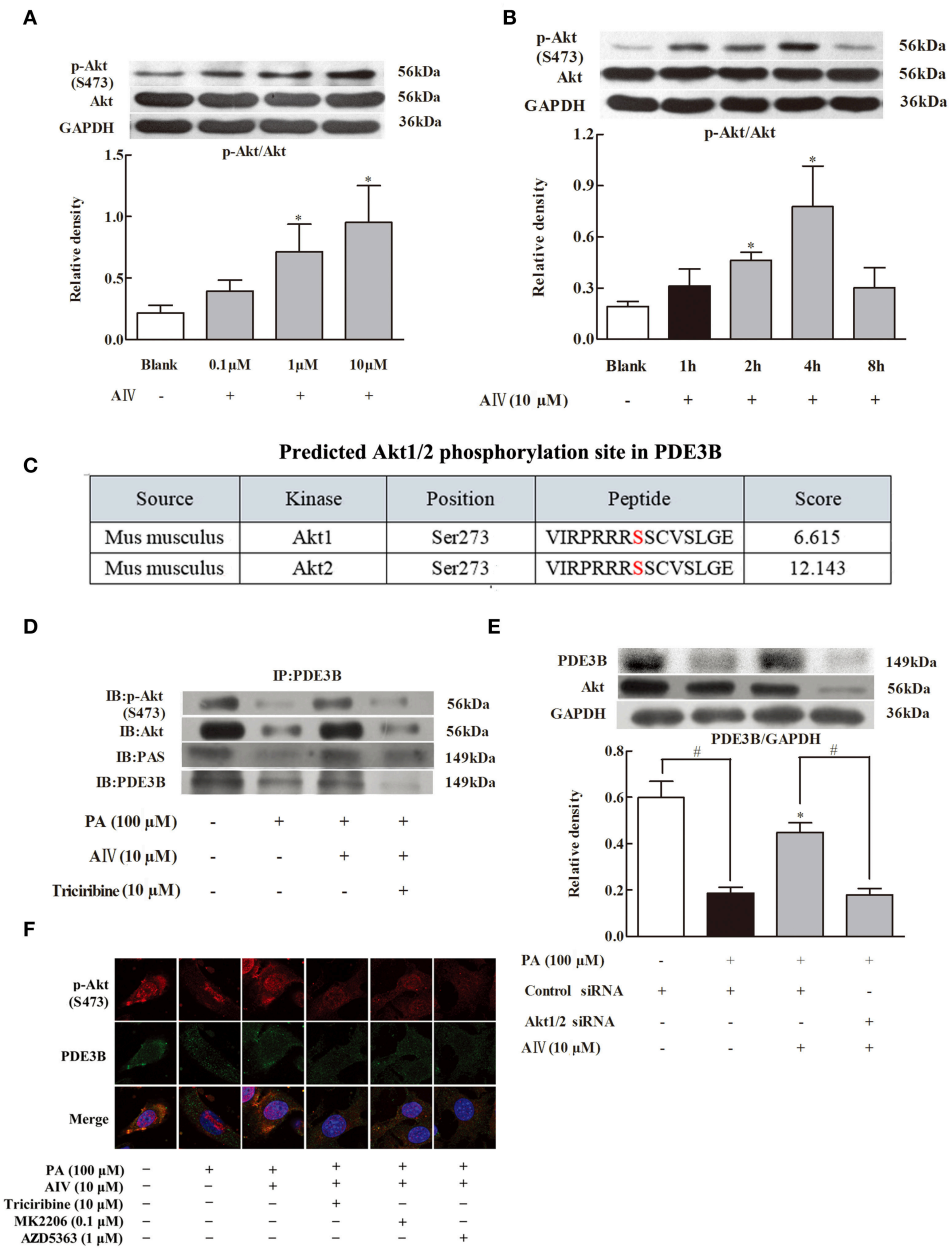
Astragaloside IV preserved PDE3B *via* regulation of Akt. **(A,B)**, Akt phosphorylation in differentiated adipocytes were examined by Western blot; **(C)**, Akt1/2 phosphorylation site in PDE3B predicted by GPS 3.0; **(D)**, p-Akt, Akt, or PAS in PDE3B in differentiated adipocytes were determined with immunoprecipitation and Western blot; **(E)**, Confocal images of PDE3B and p-Akt co-localization was observed by immunofluorescent staining. Scale bar: 10 μm; **(F)**, 3T3-L1 preadipocytes were transfected with Akt1/2 or control siRNA to silence Akt and then incubated with palmitate (PA) for 4 h. The expression of PDE3B was detected with Western blot, (Astragaloside IV, AIV; Palmitate, PA). The results were expressed as the mean ± SD (*n* = 4). ^*^p < 0.05 vs. model; ^#^*p* < 0.05 vs. control.

Next, we examined the regulation of Akt in differentiated 3T3-L1 cells and found that astrgaloside IV increased Akt phosphorylation in a concentration-dependent manner (from 0.1 to 10 μM) (Figure 3A). The result of the time course showed that astragaloside IV enhanced Akt phosphorylation in the early period and reached the highest expression at 4 h (Figure 3B). To identify whether PDE3B could be phosphorylated by Akt, complete amino-acid sequences of PDE3B were scanned by the Scansite databases. Search result revealed that mice PDE3B contains Akt consensus phosphorylation sites (Figure 3C), suggesting that the involvement of Akt in PDE3B phosphorylation. Then immunoprecipitation examination showed that phosphorylated Akt consensus sequence (PAS) presented in PDE3B and astrgaloside IV treatment increased PAS expression when cells were exposed to PA (Figure 3D), confirming the regulation of PDE3B by Akt. Meanwhile, we found that astrgaloside IV increased total Akt and p-Akt expression in PDE3B protein (Figure 3D). Consistently, immunofluorescent staining further revealed that astrgaloside IV promoted PDE3B and p-Akt co-localization against PA insults, indicating that astrgaloside IV promoted Akt translocation to PDE3B (Figure 3E). In 3T3-L1 cells, astragaloside IV preserved PDE3B protein expression, while knockdown of Akt1/2 with siRNA diminished the effect of astragaloside IV (Figure 3F), providing evidence that Akt was required for astragaloside IV to preserve PDE3B protein expression.

### Astragaloside IV suppressed lipolysis signaling and inflammation in adipose tissue of HFD-fed mice

**Figure 4:**
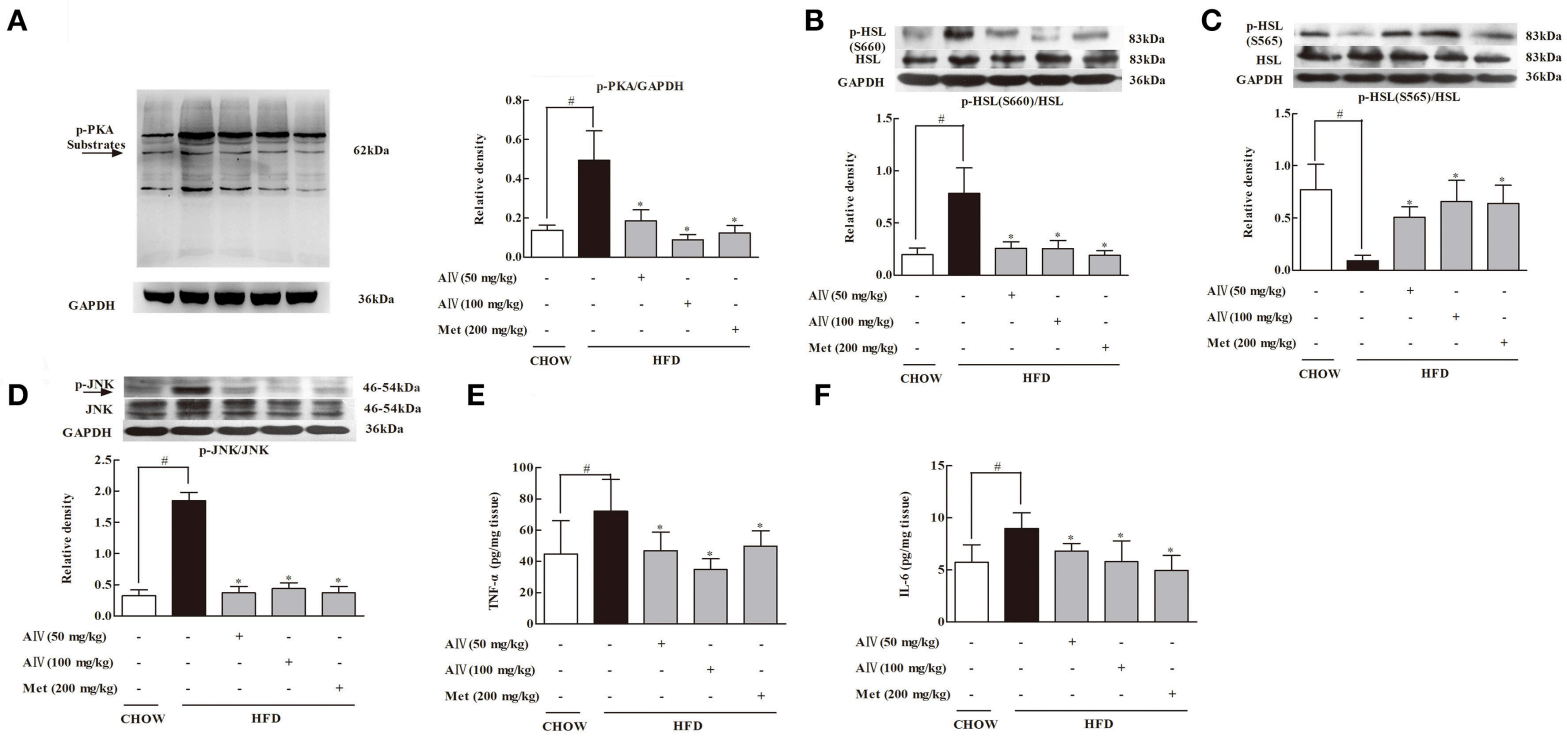
Astragaloside IV surpressed PKA/HSL signaling and inflammation in adipose tissue of HFD-fed mice. **(A)**, PKA, **(B)**, HSL(S660), and **(C)** HSL(S565) protein expressions in the adipose tissue of HFD-fed mice were examined by Western blot (*n* = 4). Arrow points to 62kDa. **(D)**, JNK protein expression in the adipose tissue of HFD-fed mice was examined by Western blot (*n* = 4). **(E,F)**, The levels of TNF-α and IL-6 in the adipose tissue of HFD-fed mice were assayed by ELISA kits (*n* = 6). (Astragaloside IV, AIV; Metformin, Met). The results were expressed as the mean ± SD. ^*^p < 0.05 vs. model; ^#^*p* < 0.05 vs. control.

PKA activation is a downstream signaling of cAMP regulation in adipose tissue. In response to cAMP accumulation, PKA 62 KD substrate phosphorylation increased in adipose tissue, a result indicating PKA activation. Astragaloside IV and metformin administration inhibited PKA activation by dephosphorylation of PKA 62 KD substrate (Figure 4A). Hormone-sensitive lipase (HSL) hydrolyzes intracellular diacylglycerol to monoacylglycerol, leading to the release of FFAs and glycerol. HSL activation is regulated by phosphorylation modification. Phosphorylation of Ser 660 activates HSL, while phosphorylation at the residue 565 is proposed to inhibit HSL activation (Djouder et al., 2010). In adipose tissue, HFD feeding increased HSL phosphorylation at Ser 660 and attenuated the phosphorylation at 565 residue, whereas these alterations were reversed by oral administration of astragaloside IV (Figures 4B,C), demonstrating its inhibitory effect on HSL activation. Similarly, metformin also effectively inhibited HSL activation.

As is known, inflammation is involved in adipose lipolysis (Rahn Landström et al., 2000; Zhang et al., 2002). In adipose tissue, HFD feeding induced JNK activation by enhancing phosphorylation and increased proinflammatory cytokine TNF-α and IL-6 production. Oral administration of astragaloside IV inactivated JNK by dephosphorylation and reduced TNF-α and IL-6 production, demonstrating its action in suppression of inflammation in adipose tissue (Figures 4D–F). Metformin also inactivated lipolysis signaling and inhibited inflammatory response in adipose tissue.

### Astragaloside IV reduced hepatic lipid deposition in HFD-fed mice

**Figure 5:**
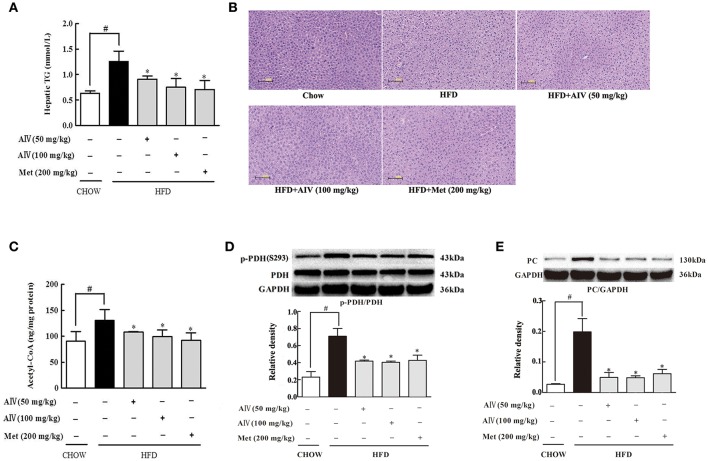
Astragaloside IV ameliorated hepatic lipid deposition in HFD-fed mice. **(A)**, Triglyceride (TG) concentration in the liver (*n* = 6). **(B)**, Hepatic H&E satining (*n* = 4). **(C)**, Acetyl CoA levels in the liver (*n* = 6). PDH phosphorylation **(D)** and PC **(E)** protein expression in the liver of HFD-fed mice was examined by Western blot (*n* = 4). (Astragaloside IV, AIV; Metformin, Met). The results were expressed as the mean ± SD. ^*^p < 0.05 vs. model; ^#^*p* < 0.05 vs. control.

Astragaloside IV inhibited adipose lipolysis and decreased the elevated levels of circulating FFAs, and this regulation should prevent ectopic lipid deposition. Indeed, astragaloside IV as well as metformin reduced hepatic lipid deposit, evidenced by reduced hepatic TG contents (Figure 5A). HE staining showed that astragaloside IV and metformin reduced hepatocellular vacuolation with respect to the control (Figure 5B). Since acetyl CoA is an end product of fatty acid oxidation, the reduced acetyl CoA by astragaloside should be a result from reduced fatty acid entry and oxidation (Figure 5C). Previously, the acetyl CoA is proposed to inhibit pyruvate dehydrogenase (PDH) activity (Sugden and Holness, 2003) with pyruvate carboxylase (PC) induction (Adina-Zada et al., 2012), contributing to gluconeogenesis. As expected, astragaloside IV improved PDH activity by dephosphorylation (Figure 5D) and reduced PC protein expression (Figure 5E) in the liver of HFD-fed mice.

### Astragaloside IV regulated akt and FoxO1 in the liver of HFD-fed mice

**Figure 6:**
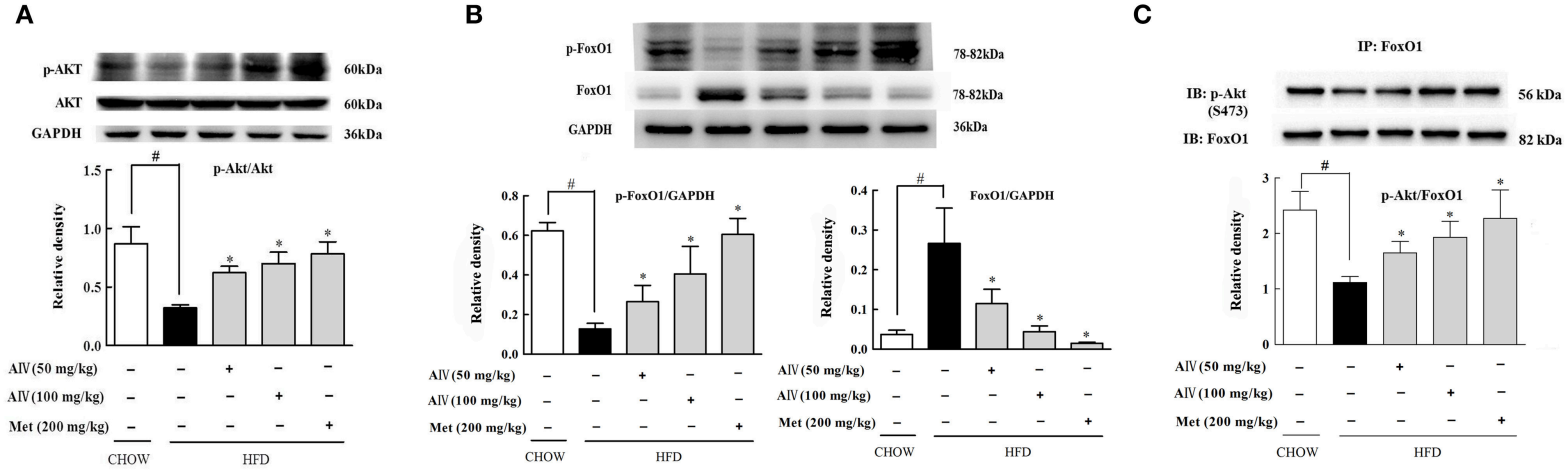
Astragaloside IV regulated Akt and FoxO1 in the liver of HFD-fed mice. **(A)**, The phosphorylation of Akt in the liver of HFD-fed mice was examined by Western blot (*n* = 4). **(B)**, p-FoxO1 and FoxO1 protein expression in the liver of HFD-fed mice was examined by Western blot (*n* = 4). **(C)**, p-Akt in FoxO1 was determined with immunoprecipitation and Western blot (*n* = 4). (Astragaloside IV, AIV; Metformin, Met). The results were expressed as the mean ± SD. ^*^p < 0.05 vs. model; ^#^*p* < 0.05 vs. control.

Astragaloside IV administration preserved Akt phosphorylation in the liver of HFD-fed mice (Figure 6A). FoxO1 is a transcription factor encoding genes for gluconeogenesis, and its activation is modulated by phosphorylation (Ozcan et al., 2012). HFD feeding increased hepatic FoxO1 protein expression with attenuated phosphorylation, indicative of FoxO1 activation, but these alternations were reversed by astragaloside IV (Figure 6B). Consistent with this, when FoxO1 protein was immunoprecipitated and then blotted using p-Akt (Ser473) antibody, astragaloside IV increased phosphorylated Akt expression in FoxO1, indicating the direct interaction between Akt and FoxO1 (Figure 6C). These results suggested that astragaloside IV suppressed FoxO1 activation *via* regulation of Akt.

### Astragaloside IV reduced hepatic gluconeogenesis

**Figure 7:**
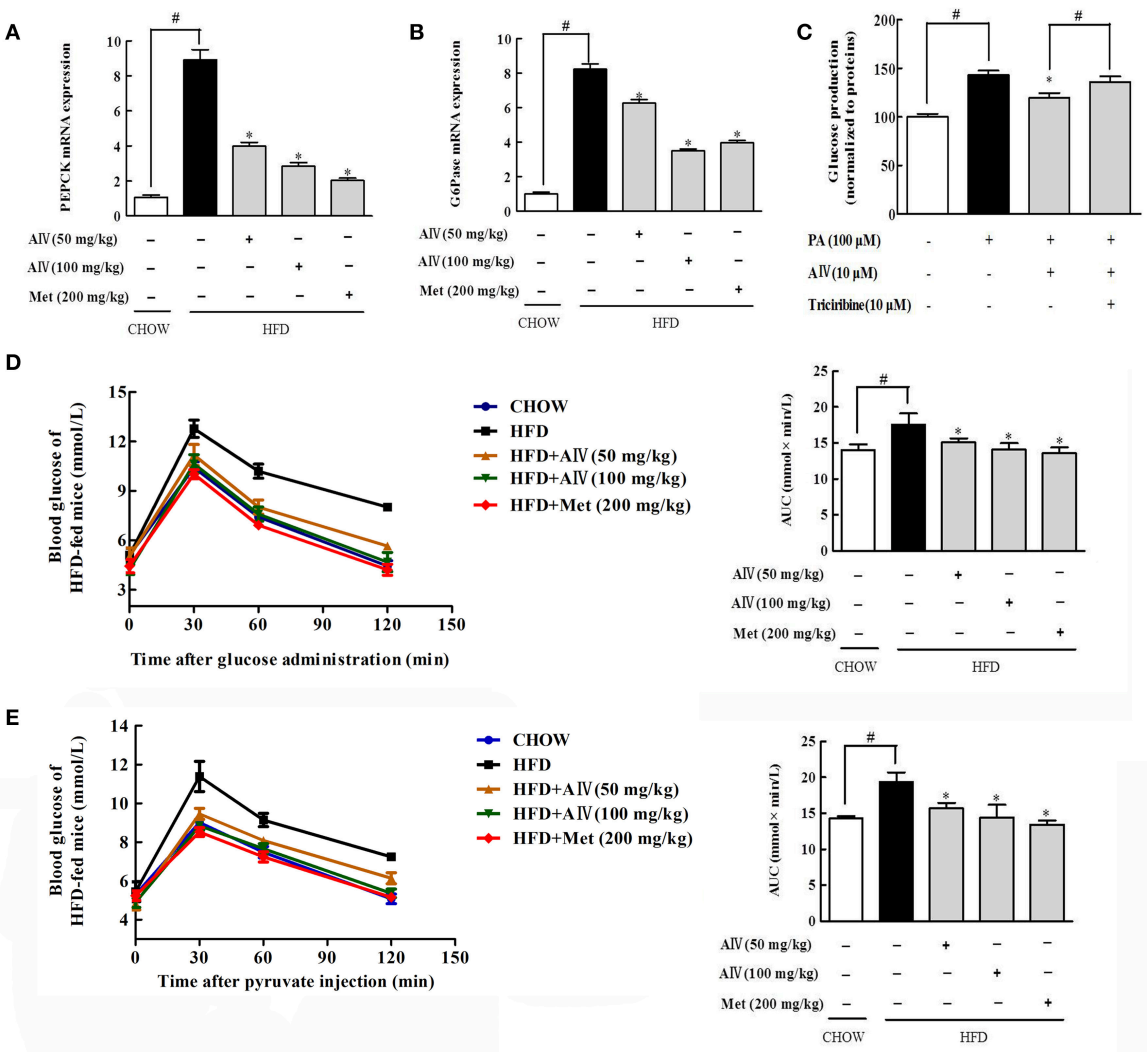
Astragaloside IV reduced hepatic gluconeogenesis. Phosphoenolpyruvate carboxykinase (PEPCK) **(A)** and glucose 6-phosphatase (G6Pase) **(B)** gene expression in the liver was examined by qPCR (*n* = 4). **(C)** Glucose production in primary hepatocytes (*n* = 6). Pyruvate tolerance test with the AUC **(D)** and glucose tolerance test with the AUC **(E)** in HFD-fed mice (*n* = 6). The results were expressed as the mean ± SD. (Astragaloside IV, AIV; Metformin, Met). ^*^p < 0.05 vs. model; ^#^*p* < 0.05 vs. control.

Phosphoenolpyruvate carboxykinase (PEPCK) and glucose 6-phosphatase (G6Pase) are gluconeogenic genes responsible for hepatic glucose production. HFD feeding induced PEPCK and G6Pase gene expression in the liver, indicative of enhanced gluconeogenesis (Figures 7A,B). Astragaloside IV suppressed gene expressions for PEPCK and G6Pase in the liver of HFD-fed mice, demonstrating the action to restrain hepatic gluconeogenesis (Figures 7A,B). As expected, astragaloside IV reduced pyruvate-driven glucose production in primary hepatocytes (Figure 7C), but the action was attenuated by Akt inhibitor triciribine. Pyruvate tolerance is employed for hepatic glucose output, because pyruvate provides the substrate for hepatic glucose production. Concordant with the inhibitory effect on hepatic gluconeogenic gene induction, astragaloside IV reduced blood glucose rise in response to pyruvate load in HFD-fed mice (Figure 7D). These results indicated that astragaloside IV restrained excessive endogenous glucose production in HFD-fed mice. Meanwhile, astragaloside IV also improved glucose tolerance in HFD-fed mice (Figure 7E).

## Discussion

Insulin inhibits adipose lipolysis through PI3K/Akt signaling. In the present study, we showed that astragaloside IV preserved PDE3B content and reduced cAMP accumulation *via* regulation of Akt in an insulin independent manner, thereby inhibiting inflammation-associated lipolysis. In view of the association of adipose dysfunction with metabolic disorder diseases (Perry et al., 2015; Ritter et al., 2015), this finding provides a mechanical explanation for the beneficial effects of astragaloside IV on the regulation of metabolic homeostasis.

In the basal state, the triacylglyceride (TG) pool in adipocytes is kept at a balance between non-esterified free fatty acids (NFFAs) release from TG and re-esterification of non-esterified fatty acids to produce TG, depending on energy demands. In obesity and diabetes, the balance is disturbed due to adipose dysfunction, leading to increased lipolysis (Greenberg et al., 2011). Indeed, we observed that adipose lipolysis increased in HFD-fed mice. Lipolysis cascade is initiated by cAMP/PKA signaling in adipose tissue. As a second messenger in response to cellular stress, cAMP is synthesized by adenylate cyclase. In adipose tissue, astragaloside IV reduced cAMP accumulation with increased AMP contents. Adenosine nucleosides are proposed to inhibit adenylate cyclase activity in fat cells (Fain et al., 1972), and consistent with this, metformin is documented to inhibit adenylate cyclase activity and reduced cAMP by elevating the levels of AMP in the liver (Miller et al., 2013). In addition, metformin has also been known to activate PDE4B in hepatocyte, contributing to the decrease of cAMP with increase of AMP level, as AMP itself is a byproducts of PDE (Johanns et al., 2016). Similar to metformin, astragaloside IV reduced cAMP generation with increased AMP contents in the adipose tissue of HFD-fed mice, suggesting that astragaloside IV could inhibit cyclase activity *via* upregulation of AMP, a regulation relative with reducing energy charge. Reduced cellular energy charge (increased AMP/ATP ratio) can induce AMPK activation. Astragaloside IV enhances AMPK activity in endothelial cells (Zhao et al., 2015), suggesting its potential to reduce cellular energy charge. In addition to the inhibition of adenylate cyclase activity by upregulation of AMP.

In adipocytes, PDE3B prevented cAMP accumulation by degradation and this regulation is influenced by the modulation of PDE3B activity. It is believed that Akt mediates the anti-lipolytic effect of insulin *via* phosphorylation of PDE3B (Kitamura et al., 1999; Frühbeck et al., 2014). In spite that Akt is proposed to be dispensable for the suppression of lipolysis by insulin (DiPilato et al., 2015; Koren et al., 2015), there is a PAS in PDE3B, indicating the potency to be regulated by Akt. Moreover, Akt exerts the ability to increase PDE3B activity by phosphorylation of PDE3B (Kitamura et al., 1999; Frühbeck et al., 2014). Therefore, it is possible that Akt could directly regulate PDE3B in an insulin independent manner. Akt traverses the cell interior with regulated localization. In response to palmitate challenge, PDE3B protein expression was reduced. Astragaloside IV promoted Akt translocation to PDE3B and induced PAS induction in PDE3B with the preserved PDE3B expression. Knockdown of Akt1/2 blocked the action of astragaloside IV to preserve PDE3B content in adipocytes when exposed to palmitate challenge, indicating that astragaloside IV protected PDE3B content in Akt dependent manner. Inflammatory molecules could impair PDE3B activity in adipose tissue and liver (Rahn Landström et al., 2000; Ke et al., 2015). In this context, the regulation of Akt dependent PDE3B activation should contribute to ameliorating adipose dysfunction inflammation involved. In the present study, pro-inflammatory cytokine TNF-α impaired PDE3B activity in the adipose tissue, indicative of the impact of inflammatory on lipolysis. Akt inhibitors diminished the protective effect of astragaloside IV on PDE3B gene and protein content, provided evidence to support the conclusion.

In response to cAMP accumulation, PKA activation modulates HSL phosphorylation to induce FFAs release. Phosphorylation of HSL occurs on multiple sites, including Ser-660, which stimulates catalytic activity, and Ser-565, which is believed to inactivate HSL (Djouder et al., 2010). As a downstream regulation from blocking cAMP/PKA signaling, astragaloside IV inactived HSL by dephosphorylation of HSL Ser-660. Meanwhile, Astragaloside IV also restored HSL phosphorylation at Ser-565, and this action should contribute to suppression of HSL activation. Inflammation is involved in adipose dysfunction, contributing to enhance lipolysis (Rahn Landström et al., 2000). Pro-inflammatory cytokine TNF-α and inflammatory signaling induce adipose lipolysis (Rahn Landström et al., 2000; Zhang et al., 2002). Astragaloside IV inactivated JNK by dephosphorylation and reduced TNF-α and IL-6 production, well demonstrating its anti-inflammatory effects. Astragaloside IV inhibited inflammation and this action should be involved in the blockage of lipolysis signaling. The anti-inflammatory effects of astragaloside IV have been well-documented (Zhang and Frei, 2015; Dong et al., 2017), our work provides evidence that the anti-inflammatory action is involved in the amelioration of adipose dysfunction.

Increased adipose lipolysis can induce ectopic fat deposits, and fat accumulation in muscle is shown to induce insulin resistance (Xiao et al., 2017b), while hepatic fat deposition enhances glucagon response to increase glucose output (Xiao et al., 2017a). Astragaloside IV decreased circulating FFAs by inhibiting adipose lipolysis, and thereby effectively prevented lipid deposition in the liver of HFD-fed mice. As a result from limited FFAs uptake by hepatocyte, astragaloside IV reduced acetyl CoA production from fatty acid oxidation, More than a metabolic intermediate, acetyle CoA is involved in the regulation of cellular metabolism (Pietrocola et al., 2015). Consistent with this, we observed increased PDH phosphorylation and PC protein expression in the liver of HFD-fed mice, a regulation likely due to acetyle CoA accumulation (Sugden and Holness, 2003; Adina-Zada et al., 2012). Pyruvate is generated in the cytoplasm through glycolysis and imported into mitochondria through mitochondrial pyruvate carrier. Mitochondrial pyruvate is converted to acetyl CoA by PDH for oxidation, or to oxaloacetate by PC for gluconeogenesis (Jeoung et al., 2014). In the present study, when HFD feeding impaired PDH activity by phosphorylation, it is reasonable to speculate that more mitochondrial pyruvate would be shifted from oxidation to gluconeogenesis pathway. Astragaloside IV improved PDH activity by dephosphorylation with suppression of PC, and this regulation should contribute to reduce hepatic glucose production by blocking substrate supply for gluconeogenesis.

Meanwhile, we also found that Astragaloside IV preserved Akt phosphorylaiton and downregulated FoxO1 protein expression with restoration of phosphorylation, indicative of FoxO1 inactivation. Astragaloside IV promoted phosphorylated Akt binding to FoxO1, providing support to the suppression of FoxO1 by Akt. As a transcription factor, FoxO1 upregulates gluconeogenic gene expression. It is well-established that Akt inactivates FoxO1 by phosphorylation to reduce hepatic glucose production. Astragaloside IV improved pyruvate intolerance in HFD-fed mice and reduced pyruvate-driven glucose production in hepatocytes, well-demonstrating the inhibitory effects of excess glucose production associated with lipid disorders. In addition to the inhibition of lipolysis *via* Akt dependent PDE3B activation in adipose tissue, these results further showed that astragaloside IV restrained hepatic gluconeogenesis through Akt-mediated FoxO1 inactivation.

**Figure 8:**
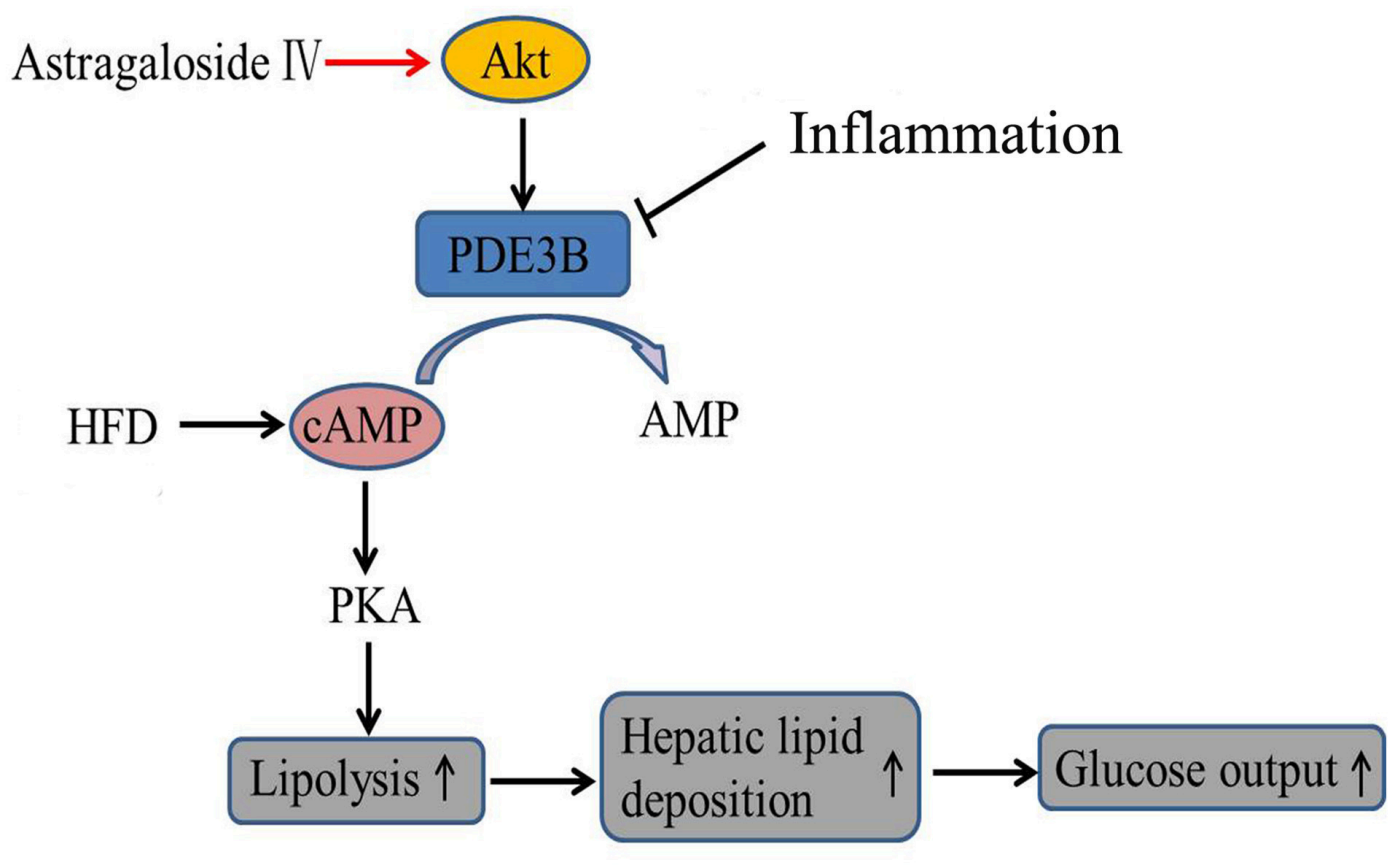
The proposed regulatory pathway for astragaloside IV action in inhibition of adipose lipolysis. Astragaloside IV (AIV) activated Akt and prevented cAMP accumulation in adipose tissue by protecting PDE3B induction from inflammation- associated impairment, and thereby inhibited lipolysis *via* blocking cAMP/PKA activation and decreased hepatic glucose production *via* reducing hepatic lipid deposition.

In conclusion, astragaloside IV enhanced Akt phosphorylation and suppressed inflammation-associated lipolysis *via* reducing cAMP accumulation in adipose tissue, and thereby reduced hepatic lipid fat deposition and restrained excess hepatic glucose production. The proposed regulatory pathway was shown in Figure 8. This finding not only elucidated a previously unrecognized role of astragaloside IV to improve adipose function, but also suggested that PDE3B activity in adipose tissue might be therapeutically targeted to ameliorate adipose dysfunction and inhibit hepatic gluconeogenesis in metabolic disorders.

## Author contributions

YL: Designed the research; QD: Performed experiments, analyzed data, and drafted the manuscript; SZ and AL: Collected data and reviewed the manuscript; BL: Contributed to the discussion and review of the manuscript. All authors approved the final version of the paper.

### Conflict of interest statement

The authors declare that the research was conducted in the absence of any commercial or financial relationships that could be construed as a potential conflict of interest.
